# Emergency Pulmonary Thromboendarterectomy in Malignancy

**DOI:** 10.1016/j.jaccas.2025.105583

**Published:** 2025-10-29

**Authors:** Manar El Bedewy, Hanad Ahmed, Daniel Sitaranjan, John Taghavi

**Affiliations:** a Università degli Studi dell'Insubria, Varese, Italy; b Department of Cardiothoracic Surgery, Royal Papworth Hospital, Cambridge, United Kingdom

**Keywords:** breast cancer, chronic thromboembolic pulmonary hypertension, embolic events, emergency surgery, patent foramen ovale, pulmonary thromboendarterectomy, right heart failure

## Abstract

**Background:**

Pulmonary thromboendarterectomy (PTE) is the treatment of choice for operable chronic thromboembolic pulmonary hypertension, and it is typically planned electively after thorough diagnostic evaluation.

**Case Summary:**

We present the case of a 49-year-old woman with breast cancer who underwent an emergency PTE after presenting with acute-on-chronic pulmonary embolism, acute right ventricular dysfunction, and a thrombus-in-transit via a patent foramen ovale.

**Discussion:**

Emergency PTE is rarely undertaken owing to complexity, the need for deep hypothermic circulatory arrest, and the high perioperative risk. Moreover, patients with active cancer are generally considered poor surgical candidates. We therefore report this case of an emergency PTE and patent foramen ovale closure in a hemodynamically unstable patient with breast cancer. To the best of our knowledge, this has not been previously documented in the literature.

**Take-Home Message:**

Emergency PTE can be an effective intervention in selected patients with active malignancy.

## Graphical Abstract

**Figure:**
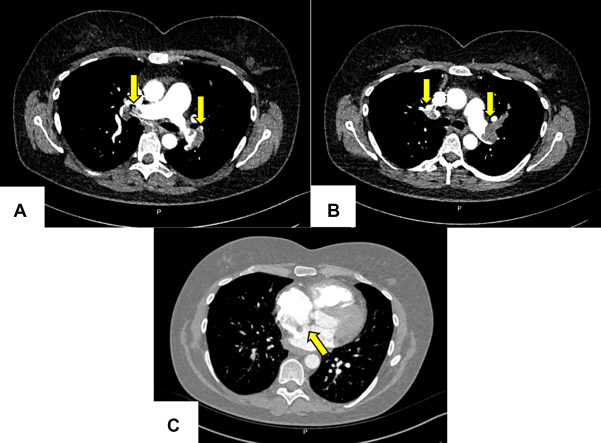

## History of Presentation

A 49-year-old woman with breast cancer who was undergoing chemotherapy presented to her local hospital with acute shortness of breath and chest pain, which rapidly progressed to hemodynamic instability. Imaging revealed multiple acute pulmonary emboli (PEs), with signs of right heart strain, a patent foramen ovale (PFO), and a thrombus-in-transit across the PFO. The patient was urgently transferred to our center for further surgical management.

**Take-Home Message**

• Emergency PTE can be an effective intervention in selected patients with active malignancy.

## Medical History

The patient's medical history included deep vein thrombosis and PE nearly 2 decades earlier, for which she was no longer receiving anticoagulation. These events were associated with the use of oral contraceptives. She was also undergoing active treatment for a stage 2 multifocal right-sided breast cancer, with no evidence of metastasis. A biopsy revealed a grade 3 ER-positive, HER2-negative invasive ductal carcinoma. Chemotherapy was initially planned to include epirubicin and cyclophosphamide followed by paclitaxel. Given her acute presentation, chemotherapy was interrupted and limited to 3 cycles of epirubicin and cyclophosphamide, with the last cycle occurring nearly a month before presentation to our center.

## Differential Diagnoses

Considerations included acute-on-chronic pulmonary embolic disease and a thrombus-in-transit across a PFO.

## Investigations

**Figure 1:**
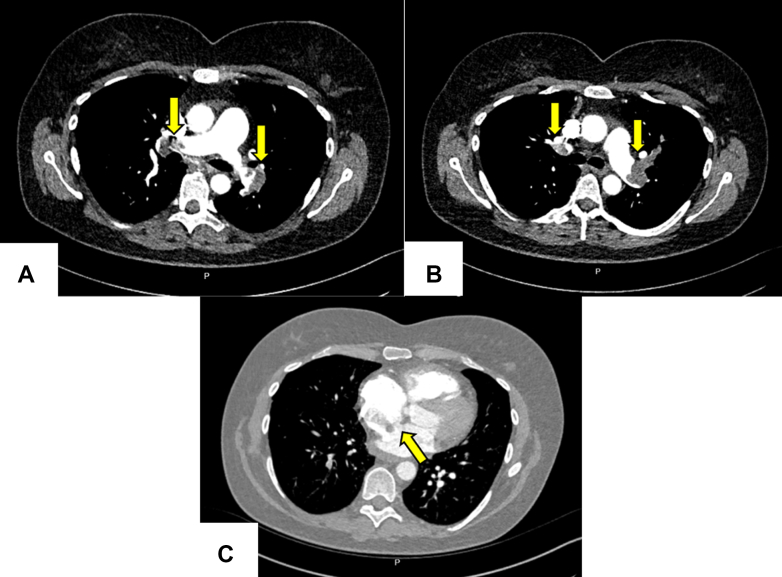
Initial Findings on Computed Tomography (A) The occlusive thrombus in the right and left main pulmonary arteries is shown just before their division (arrows). The main pulmonary artery is clear of thrombus. (B) The occlusive thrombus in the left main pulmonary artery is shown (arrows) extending to totally occlude the left upper lobe branch. (C) The patent foramen ovale is shown, with thrombus visible in the right atrium (arrow).

Computed tomography demonstrated significant thromboembolic burden, with signs of right heart strain and a curvilinear filling defect extending from the right atrium to the left atrium through a PFO (Figure 1). This finding was confirmed by preoperative transthoracic echocardiography (Videos 1 and 2). In the operating room, transesophageal echocardiography revealed a markedly dilated and functionally impaired right ventricle. The thrombus across the PFO was also no longer present.

## Management

**Figure 2:**
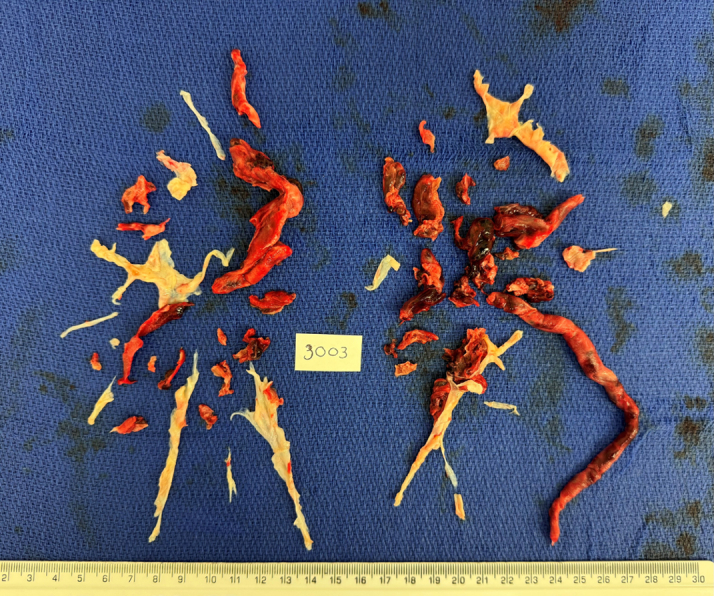
Pulmonary Thromboendarterectomy Specimen

Given the imminent risk of embolic complications and severe right heart failure, emergency surgery was performed. After induction of general anesthesia, the patient became tachycardic and hypotensive. Surgical intervention included exploration of cardiac chambers, embolectomy, and progression to full pulmonary thromboendarterectomy (PTE) and PFO closure. No thrombus was found in the atria or ventricles. Acute thrombus with underlying organized material was found in the right pulmonary artery, consistent with type 2 chronic thromboembolic pulmonary hypertension (CTEPH). Similar findings were present in the left pulmonary artery. The PTE specimen is demonstrated in Figure 2.

## Outcome and Follow-Up

**Figure 3:**
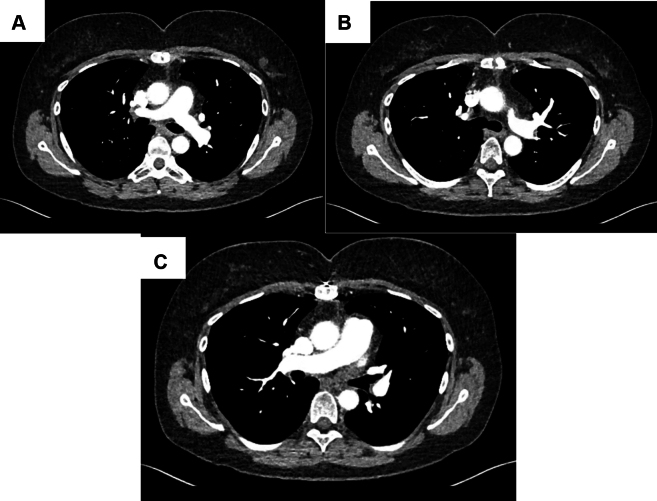
Postoperative Computed Tomography Images Postoperative follow-up computed tomography illustrating disease-free opacification of the main pulmonary artery and its proximal branches, in contrast to Figure 1.

Postoperative recovery was complicated by new-onset atrial fibrillation, which was reverted with amiodarone. The patient was discharged on oral anticoagulation on postoperative day 7, walking independently without supplemental oxygen. At the 3-month follow-up, she had fully recovered, with baseline exercise tolerance. Echocardiography showed normal left and right ventricular function and a repaired PFO (Video 3). Post-operative CT illustrated disease free opacification of the pulmonary arteries (Figure 3). Histopathology revealed subacute and chronic thrombus with luminal fibrosis and recanalizing vessels, consistent with chronic disease. There was no evidence of vasculitis or tumor. The patient is now awaiting breast cancer surgery and is on hormone therapy with Zoladex (goserelin) and letrozole. She has not received additional chemotherapy beyond the initial 3 cycles.

## Discussion

PTE is the gold standard surgical treatment for operable CTEPH, a condition caused by persistent obstruction of the pulmonary arteries due to fibrotic thromboembolic material and vascular remodeling.^1^, ^2^, ^3^, ^4^ It is typically performed electively, after detailed investigations to ensure appropriate patient selection, and it offers significant improvement in pulmonary hemodynamics, functional capacity, and survival.^5^^,^^6^ There are few comprehensive studies in the current literature describing the indications, safety, and outcomes of patients undergoing emergency PTE, particularly in the context of active malignancy, highlighting an important knowledge gap.

In this patient, right heart failure and the presence of a thrombus straddling a PFO created an urgent clinical scenario given the risk of paradoxical and systemic embolism.^4^ Only a few cases of urgent PTE have been reported in the literature, with no documented emergency cases in the context of a PFO. Viana et al^7^ reported 2 cases of acute hemodynamic decompensation due to acute-on-chronic PE, refractory to thrombolysis and managed with inferior vena cava filters. Although the timing of PTE was not clearly specified, surgical intervention was undertaken after a trial of thrombolysis and inferior vena cava filter insertion. More recently, Jung et al^8^ described the case of a young female patient who underwent PTE for persistent hemoptysis in the context of acute-on-chronic PE without right ventricular dysfunction. None of the previously reported cases involved patients with malignancy.

Patients with cancer are at increased risk of both arterial and venous thromboembolism. This risk is further influenced by the cancer's site and stage, as well as the chemotherapy treatment.^9^ Platinum-based agents such as carboplatin and anthracyclines such as doxorubicin are known to be associated with a higher risk of venous thromboembolism.^9^ The precise mechanisms by which chemotherapy promotes thrombosis are not fully understood, and it remains difficult to disentangle these effects from the baseline prothrombotic state associated with cancer itself. Proposed mechanisms include chemotherapy-induced endothelial dysfunction, increased tissue factor expression, aberrant platelet activation, and disruption of natural anticoagulant pathways.^9^ These complications can be life-threatening, and—as this case illustrates—may result in PE, acute right ventricular failure, and imminent embolic events.

Patients undergoing PTE for CTEPH in the setting of active cancer have been shown to have worse outcomes, with 5-year survival rates significantly lower than those in patients without active cancer or in cancer-free cohorts.^10^ Nonetheless, this case demonstrates that favorable outcomes can be achieved in selected patients with active malignancy and perioperative chemotherapy.

## Conclusions

This case demonstrates that, in carefully selected patients, emergency PTE may provide a lifesaving strategy, even in the presence of active malignancy and hemodynamic instability. Although emergency PTE remains uncommon given its technical demands and perioperative risk, successful outcomes may be achieved in high-volume centers with specific expertise in pulmonary vascular surgery.

## Funding Support and Author Disclosures

The authors have reported that they have no relationships relevant to the contents of this paper to disclose.

## Appendix

Video 1 Transthoracic Echocardiogram Demonstrating Thrombus-in-Transit via PFO

Video 2 Close-Up Demonstrating Thrombus-in-Transit via PFO

Video 3 Postoperative Follow-Up Transthoracic Echocardiogram
